# Topics of Public Interest

**Published:** 1904-11

**Authors:** 


### TOPICS OF PUBLIC INTEREST.

### Vaccination in the Schools.

THE Court of Appeals has affirmed the right of educational authorities to exclude from the public schools all children who have not been vaccinated. The case on which the decision was written was that by Edward C. Viermeister, of Brooklyn, against the board of education to compel the reception of his child in the public schools in spite of the declination of the parents to permit the child to be vaccinated. The lower court decided against Viermeister and he appealed the case. The arguments were made before the Court of Appeals and on October 18, 1904, a decision was handed down affirming the judgment of the lower court.

This makes the position of school and health authorities plain. As soon as a certified copy of the decision reached Buffalo, the health commissioner gave out an interview in which he stated that hereafter no child would be admitted to the public schools who was not either vaccinated by the physicians sent for that purpose to the schools by the health department, or presented a certificate of successful vaccination from the family physician.

The Viermeister case had an interesting phase in that it was based on the constitution which gives every child opportunity to secure a common school education.

In Ohio there is a similar case before the courts. On October 10, 1904, Charles M. Rifer, of Barberton, brought suit against the board of education for an alternative writ of mandamus to compel the reception of his four children who had been excluded from the schools because they had not been vaccinated. This case whatever its termination in the lower courts will be carried up, and now that the New York Court of Appeals has rendered a decision so sweeping and so uncompromisingly in favor of public health and protection, it is more than likely the decision will be used as a precedent for the handing down of a similar decision when the Ohio case reaches the highest court.

---

### A Modern Crusade.

THE Illinois State Board of Health is engaging in a vigorous and commendable crusade to stamp out tuberculosis from the state. For some time the board has been sending out a leaflet to the public on the prevention and cure of consumption. A third revised edition of this has just been issued and it has been increased to a popular monograph of twenty-four pages, entitled, "The Cause and Prevention of Consumption." On the cover is this:

"The Illinois State Board of Health, as created by law, is charged with the general supervision of the interests of the health and life of the people of the state. In view of the fact that consumption, a dangerously communicable disease, is causing more human suffering and greater loss of life among our people than any other existing disease, it becomes the duty of the board to warn the people of its dangers and to advise them of the measures necessary to prevent its spread. Should this circular be the means of saving one human life,—and if the directions enjoined herein be followed, it will save many thousands,—the State Board of Health will feel that its work has been well done."

The work treats on how to avoid consumption, how to prevent its spread, how to destroy sputum, hygiene of the sick room, consumption and schools, climatic conditions, and the like. Consumptives are urged to be careful regarding sputum. All through the book "no spit, no consumption," is impressed on the reader. The following advice is given on the avoidance of consumption:

"The important points in the prevention of consumption are: pure air, sanitary surroundings as abundance of light and fresh air in the dwellings, office and workshop, proper clothing, good food properly cooked, moderate rest and recreation, avoidance of all excesses; in other words moderate living. The excessive use of alcoholic liquors lowers vitality, favors infection and hastens a fatal termination."

Regarding treatment we quote:

"Live 'out-of-doors' day and night, winter and summer. Wear proper clothing. Have no fear of night air and none of draughts. Court the sunshine. Avoid damp houses or rooms. Avoid crowds, smoke and dust. Avoid all excesses. Be careful that you do not excercise when you should rest. Eat plenty of good nourishing food. Drink plenty of good water. Keep your body clean. Take no drugs except on the advice of a physician. Never swallow the sputum which you hawk or cough up. Be hopeful and cheerful."

If a copy of this pamphlet could be placed in the hands of every individual in the United States, there would immediately be a great reduction in the amount of consumption in this country and eventually it could be practically eradicated. The Illinois Board supplies the monograph free to all residents of Illinois and is making special effort to get it into the hands of consumptives. This work is under the supervision of Dr. Jas. A. Egan, of Springfield, secretary of the board, who is one of the ablest and most energetic sanitarians of the day. The Illinois Medical Society has also recently established at Ottawa, Ill., a colony for the treatment of consumptives, modeled after institutions in the east, under the charge of Dr. J. W. Pettit. Illinois is to be congratulated on having a profession working so energetically and unselfishly for the public welfare.

This is but another demonstration of the fact that the medical profession works for the public good regardless of its own interests. In spite of the stale newspaper jokes about doctors, medical men are working for public health which means cutting off their own revenues. Although unappreciated by the general public the medical profession is laboring to bring about better methods of living and less disease. The time is coming when there will be less work for physicians. Medical societies, instead of meeting to arrange prices and further selfish ends, are gathering to promote the public health and reduce their source of income. How many other occupations and societies are doing likewise?—*Wisconsin Medical Recorder.*

---

### The Crusade Against Tuberculosis.

THE following circular has been issued from the Board of Health office:

BUFFALO, October 1, 1904.

This communication is sent out in the hope and expectation of enlisting the active coöperation of the members of the medical profession in a determined and sustained effort to combat the spread of tuberculosis. Buffalo must not be behind in the crusade which is at present being carried on throughout this and nearly all civilised countries.

Since the discovery of the specific germ of tuberculosis and the recognition of its spread through the sputum, the possibility of controlling the disease, perhaps of completely stamping it out, has attracted the attention of medical men. The result has been a widespread movement, with the object of minimising, or possibly exterminating the disease which is responsible for nearly one-seventh of human mortality.

As a means to this end, the education of the public, and especially of consumptives, is clearly all important. The active, earnest coöperation of physicians will be a necessary factor in making this most important movement a complete success. Every physician is urged to renewed activity in instructing the public generally, in availing himself of the privilege of the free examination of sputum, in reporting all cases as soon as discovered, and in furthering the efforts of the department to control the disposition of the sputum and the disinfection of the patient, his clothing and domicile.

That these matters are of the greatest importance to public health, and that they demand enforcement, even when involving some slight annoyance perhaps to the patient, must be evident to all fair-minded, progressive physicians.

The department of health is glad to announce that the tenement and tuberculosis committee of the charity organisation society is actively interested in this work and proposes to supplement the work of the department by means of public addresses, by newspaper articles, the distribution of literature, and the like. The coöperation of the department of public instruction, as well as of labor unions and other bodies, is in prospect.

This is a great work and one full of promise. It can be made a success only by the active, earnest efforts of the medical profession, which has here an opportunity to render the community a great service and one which will not fail to redound to its credit.

WALTER D. GREENE, M. D.,
*Health Commissioner.*

P. W. VAN PEYMA, M. D., GEORGE W. GILLETTE, GIOVANNI BANCHETTI, FREDERIC ALMY, JOHN D. LARKIN, JR., HENRY BULL,
*Committee of the Charity Organisation Society.*

---

### The Pneumonia Commission of the New York Board of Health.

THIS board met for the first time Tuesday evening, October 18, 1904. Dr. Edward G. Janeway, of New York, was elected president and Dr. T. Mitchell Prudden, of New York, secretary. After a general discussion the commission decided to make a systematic study of pneumonia in New York, taking into consideration the frequency and variation of occurrence, evidence of communicability, mouth infection, seasonal relations, collection of statistics, and geographical relations. The commission was subdivided into a clinical committee and a bacteriological committee. The former is composed of Drs. Janeway, Osler, Musser, Billings, and Holt; the latter of Drs. Welch, Prudden, and Smith. Dr. Biggs will serve on both committees.—*N. Y. Medical Record.*

